# Interactive Effects of Exercise, Sex Hormones, and Transient Congenital Hypothyroidism on Long-Term Potentiation in Hippocampal Slices of Rat Offspring

**DOI:** 10.32598/bcn.9.10.170

**Published:** 2019-03-01

**Authors:** Leila Derafshpour, Ehsan Saboory, Abbas Ali Vafaei, Ali Rashidy-Pour, Shiva Roshan-Milani, Yousef Rasmi, Yousef Panahi, Hamidreza Sameni

**Affiliations:** 1. Laboratory of Learning and Memory, Research Center of Physiology, Semnan University of Medical Sciences, Semnan, Iran.; 2. Neurophysiology Research Center, Urmia University of Medical Sciences, Urmia, Iran.; 3. Cellular and Molecular Research Center, Urmia University of Medical Sciences, Urmia, Iran.; 4. Department of Basic Science, Faculty of Veterinary Medicine, University of Tabriz, Tabriz, Iran.; 5. Nervous System Stem Cells Research Center, Semnan University of Medical Sciences, Semnan, Iran.

**Keywords:** Exercise, Hypothyroid, Sex hormones, Long-Term Potentiation

## Abstract

**Introduction:**

The long-term adverse effects of transient thyroid function abnormalities at birth on intellectual development are proven. The effect of exercise increases in the presence of sex hormones. The current study aimed at investigating the possibility that a combination of sex hormones and exercise has synergistic effects on neural plasticity in Transient Congenital Hypothyroidism (TCH) rats.

**Methods:**

To induce hypothyroidism in the mothers, Propylthiouracil (PTU) was added to drinking water (100 mg/L) on the 6th day of gestation and continued until the 21^st^ Postnatal Day. From Postnatal Day (PND) 28 to 47, the female and male pups received 17β-estradiol and testosterone, respectively. The mild treadmill exercise began 30 minutes after the sex hormones or vehicle administration. On PND 48, electrophysiological experiments were performed on brain slices.

**Results:**

Increase of Long-Term Potentiation (LTP) was observed in sedentary-non-hormone female rats of TCH group, compared with that of the control. The exercise enhanced LTP in control rats, but the hormones showed no significant effect. The effect of exercise and sex hormone was not significant in the TCH group. The combination of exercise and testosterone enhanced LTP in TCH male rats, while the combination of exercise and estradiol or each of them individually did not produce such an effect on LTP in TCH female rats.

**Conclusion:**

The study findings showed an increase in excitatory transmission despite the returning of thyroid hormone levels to normal range in TCH female rats. Also a combination treatment including exercise and testosterone enhanced LTP in male rats of TCH group, which was a gender-specific event.

## Highlights

- Long-Term Potentiation (LTP) was facilitated in Transient Congenital Hypothyroidism (TCH) male rats treated with exercise and testosterone.
- Exercise and testosterone individually did not show any effect in TCH male rats.
- Combination of exercise and estradiol had no effect on LTP in TCH female rats.
- Exercise and estradiol individually had no effect on LTP in TCH female rats.

## Plain Language Summary

Because of the increased survival of many premature infants, the prevalence of transient thyroid dysfunctions has increased, too. These abnormalities can result in lower intelligence in adulthood. Decreased plasma concentrations of both testosterone and estradiol are observed in hypothyroidism that can be one of the causes of cognitive failure booster. In our study, hypothyroidism was induced by propylthiouracil from the 6th day of gestation until the 21st postnatal day in rats. From postnatal day 28 to 47, the female and male pups received 17β-estradiol and testosterone, respectively with training exercise. On postnatal day 48, electrophysiological experiments were performed. The combination treatment of exercise and testosterone were effective in male rats with transient congenital hypothyroidism group but not in female rats.

## 1. Introduction

Thyroid hormones are essential for the normal development of the central nervous system (Williams, 2008). During normal gestation, thyroid hormones respond to the increased physiologic demands of the growing fetal placental unit (Klubo-Gwiezdzinska, Burman, Van Nostrand, & Wartofsky, 2011). Reductions in myelination, impairments in proliferation and migration of cells, and the retardation of synapse formation are the results of thyroid hormones deficiency during brain development (Bernal, 2002). Congenital Hypothyroidism (CH) occurs during the fetal and/or neonatal period. CH is associated with a risk of brain damage and mental retardation (Calaciura et al., 1995) and is divided into two main groups of permanent and transient. Transient Congenital Hypothyroidism (TCH) is defined as a transient abnormality of the thyroid function in newborns, which later reverts to normal (Bhavani, 2011).

The prevalence of transient thyroid function abnormalities has increased due to the increased survival of many premature infants (Bhavani, 2011). According to research, these transient disorders reduce about 10–15 Intelligence Quotient (IQ) points in school-age children with euthyroid in areas with severe endemic goiter compared with those of the controls (Bleichrodt et al., 1996). At the age of 6–9 years, children born and lived in areas with iodine deficiency that showed biochemical signs of abnormal thyroid function at birth had a lower IQ than the matched controls that had normal thyroid function at birth and lived in the same environmental conditions. The TCH group have lower global, verbal, and performance IQs compared with the normal group (Calaciura et al., 1995).

It is known that the maintenance of neuronal performance and protection against damage can be influenced by both endogenous and exogenous factors. Among the former, gonadal steroid hormones seem to be potent bio-modulators, while exercise highlighted as an exogenous factor. During the prenatal period, gonadal steroid hormones are essential for the development of the central nervous system, the organization of neural circuits, and the modulation of synaptic plasticity and neuroprotection, particularly in the hippocampus (Collaer & Hines, 1995; Fester et al., 2011; Haimov-Kochman & Berger, 2014; McEwen, 2001).

It is claimed that there are connections between endocrine secretions of the adrenal, thyroid, and sex glands (Korenchevsky & Hall, 1941) and it is well-established that hypothyroidism disrupts reproductive functions in many species, and thyroidectomy results in the decrease of basal levels of testosterone in male rats (Chiao et al., 1999) and the rats subjected to transient neonatal hypothyroidism have consistently low levels of plasma testosterone (Maran et al., 2000). Also, decreased plasma concentrations of both testosterone and estradiol are observed in females with hypothyroidism (Krassas, 2000). TCH is associated with decreased levels of testosterone (Maran et al., 2000) and estrogen (Talsness et al., 2008), in turn, it can be one of the causes of cognitive failure booster.

Exercise has beneficial effects in young adult animals (Berchtold, Castello, & Cotman, 2010). In rodents, the effects of wheel running and treadmill training have a substantial impact on the improvement of spatial learning (Liu et al., 2009). In addition, exercise can increase cognition ability through hippocampal neurogenesis. Moreover, exercise enhances synaptic plasticity, neuro-transmission, and growth factor gene expression in the hippocampus of physically active rats and mice (van Praag, Shubert, Zhao, & Gage, 2005).

It is a known fact that the activity of numerous glands and the production of their hormones are affected by exercise (Ciloglu et al., 2005). Research results indicate that exercise-associated benefits in discrimination learning are removed in the absence of gonadal hormones. Thus, an improvement in discrimination learning was observed in rats with circulating gonadal hormones, showing that there may be an interaction between gonadal hormones and exercise that initiated this enhancement (Eddy, Rifken, Toufexis, & Green, 2013). The thyroid is also affected by exercise (Ciloglu et al., 2005). Besides the metabolic effects of exercise on thyroid hormones, treadmill exercise during the postnatal period in hypothyroid rat pups results in the improvement of short-term memory and spatial learning ability (Shin et al., 2013).

It is known that hippocampus is a brain region involved in cognitive skills such as learning and memory (Gerges & Alkadhi, 2004). Memory is encoded by the modification of synaptic strengths such as Long-Term Potentiation (LTP) (Nabavi et al., 2014). LTP is a model of activity-dependent synaptic plasticity noticeably observed in the hippocampus and is also the cellular mechanism for learning and memory (Li et al., 2012; Whitlock, Heynen, Shuler, & Bear, 2006). Also, thyroid receptors are present in the hippocampus, an area involved in learning and memory. Consequently, It is reported that changes in thyroid hormone levels can disrupt hippocampal-associated learning and memory (Shafiee, Vafaei, & Rashidy-Pour, 2016), synaptic plasticity (Gilbert, 2004), and neurogenesis (Shin et al., 2013). Iodine deficiency and hypothyroidism during critical periods of brain development impair LTP induction in the hippocampus (Dong et al., 2005) and enhance the expression of LTP in the hippocampal slices in rats that undergo exercise training (Nabavi et al., 2014) and the ones treated with 17β estradiol (Li et al., 2012) and testosterone (Smith, Jones, & Wilson, 2002).

The current study hypothesized that the beneficial effects of exercise may not be apparent in conditions such as low level of sex hormones in TCH rats. It was also hypothesized that the exercise and sex hormones effects can be synergistic in the enhancement of LTP. The effect of combined exposure to physical activity and sex hormone treatment on LTP in TCH rats is not explored yet. Therefore, the current study aimed at examining whether exercise, combined with the administration of sex hormones, might exert different effects compared with hormone therapy alone.

## 2. Methods

### 2.1. Ethical approval

All experimental protocols were undertaken in accordance with the guidelines of the 1975 Declaration of Helsinki, as reflected in the guidelines of the Medical Ethics Committee, Ministry of Health, Iran. In addition, the Regional Medical Ethics Committee in West Azerbaijan Province, Iran, approved the study.

## 2.2. Subjects

Male and female Wistar rats (200–250 g) at 10 weeks of age were obtained (Animal Facility of Urmia University of Medical Sciences, Urmia, Iran) and used. Animals were housed under controlled conditions (12:12-hour light/dark cycle; lights on 07:00 am; temperature at 22±2°C) and provided with food and water. All female rats were mated at 12 weeks with a male and checked daily for the presence of a vaginal plug. If a plug was located, the female rat were immediately transferred to a new cage (Ebrahimi, Saboory, Roshan-Milani, & Hashemi, 2014; Hashem, Ebrahimi, Saboory, & Roshan-Milani, 2013; Tavassoli et al., 2013). Pregnant rats were divided into control and TCH groups. Chemical treatment started on the 6^th^ day of gestation (GD 6) and continued until the 21^st^ Postnatal Day (PND 21).

The TCH group received 100 ppm of the antithyroid drug Propylthiouracil (PTU) (Gottesfeld, Butler, & Findley, 1985) (Sigma, St. Louis, MO, USA) added to their drinking water (Lasley & Gilbert, 2011) while the control group consumed tap water. After weaning, the off-spring of the control and TCH rats were divided into four subgroups for each gender. All subgroups consisted of six rats of each gender, housed with free access to food and water. When the PTU treatment was terminated on PND 21, two pups from each dam were rapidly decapitated. Trunk blood was collected for analysis and measuring the total T4 and serum thyroid-stimulating hormone (TSH) after the centrifugation (3000 g, 10 minutes) of clotted samples, blood serum was separated and stored at −80°C. On PNDs 28–47, exercise protocols and gender-specific hormone administration were conducted. On PND 48, trunk blood was collected for measuring serum TSH and total T4. Electrophysiological experiments were also performed.

### 2.3. Experimental groups

The control and TCH rats were randomly assigned into four subgroups per gender (n=6 rats of each gender per subgroup); i.e. six sub-groups in total: sedentary-control-non-hormone (SED/CON/NH), sedentary-control-hormone (SED/CON/H), treadmill exercise-control-non-hormone (TE/CON/NH), treadmill exercise-control-hormone (TE/CON/H), sedentary-hypothyroid-non-hormone (SED/HYP/NH), sedentary-hypothyroid-hormone (SED/HYP/H), treadmill exercise-hypothyroid-non-hormone (TE/HYP/NH), and treadmill exercise-hypothyroid-hormone (TE/HYP/H).

### 2.4. Exercise protocols

Rats in sub-groups TE/CON/NH, TE/CON/H, TE/HYP/NH, and TE/HYP/H ran 30 minutes, once a day (at 12:00 pm) on a leveled motorized treadmill for 20 consecutive days with the speed from 2 m/minute for the first five minutes, 5 m/minute for the following five minutes, and 8 m/minute for the last 20 minutes, and gradient of zero. These settings represent a low-intensity treadmill exercise. The rats in subgroups SED/CON/NH, SED/CON/H, SED/HYP/NH, and SED/HYP/H were left on the treadmill without running for the same period as the rats in the exercise group (Kim et al., 2010). Exercise began 30 minutes after the subcutaneous (SC) injection of sex hormones or vehicle administered.

### 2.5. Treatments

In subgroups SED/CON/H, TE/CON/H, SED/HYP/H, and TE/HYP/H, male rats received (SC) 0.5 mg/kg of testosterone and female rats received (SC) 5 μg/kg of 17β-estradiol (Sigma, St. Louis, MO, USA) daily, for 20 consecutive days. To prepare the drugs, at first, estrogen and testosterone were dissolved in alcohol, and then distilled water was added to a concentration of 3% alcohol. The rats in subgroups SED/CON/NH, TE/CON/NH, SED/HYP/NH, and TE/HYP/NH were injected with 2 mL/Kg of vehicle (5.8 mL of distilled water +0.2 mL of 96% ethanol). The volume of injection was similar for all the rats.

### 2.6. Electrophysiological experiments

#### 2.6.1. Slice preparation

Electrophysiological experiments were performed on PND 48. Rats were anesthetized with diethyl ether and subsequently decapitated. The brains were removed and bathed in ice-cold (2–4°C) standard artificial cerebrospinal fluid (ACSF) that was continuously gassed (mixture of 95% O_2_/5% CO_2_). The composition of ACSF solution (in mM) was as follows: 123 NaCl, 2.5 KCl, 1.5 CaCl_2_, 2 MgSO_4_, 25 NaHCO_3_, 1.2 NaH_2_PO_4_, and 10 glucose. Transverse slices (450 μm) were prepared with a Vibroslice (Campden Instruments, Silbey, UK). The slices were stored in a brain slice keeper oxygenated with 95% O_2_/5% CO_2_ at room temperature for at least one hour before use. After recovery, a single slice was laid in the recording chamber and perfused constantly with ACSF at the rate of 2 mL/minute and at 30–33°C (Panahi et al., 2017). In the current study, one slice per rat was used and recorded successfully.

#### 2.6.2. Electrophysiological recording

A bipolar stimulating electrode was used to stimulate the activities of extracellular Schaffer collaterals in the stratum pyramidal of CA1 region. Synaptic transmissions were recorded by a glass microelectrode (2–6 mΩ) filled with 150 mM NaCl to record field Excitatory Post-Synaptic Potentials (fEPSP) and Population Spikes (PS). Fifteen minutes after electrode placement, stimuli were delivered using an isolator (Data Acquisition Science-Beam-D3111, Tehran, Iran) composed of constant current rectangular stimulus pulses of 200 μs, 10–250 μA, 0.1 Hzm and recording signals were filtered (1 to 3 KHz band pass) and amplified by an amplifier (×1000) (DAM 80, WPI, Sarasota, FL, USA). Signals were passed through an analog-to-digital interface (Data Acquisition ScienceBeam-D3111) to a computer, and data were analyzed using potentialize 2.05 (ScienceBeam).

Baseline response was recorded for 30 minutes with the stimulation frequency of 0.1 Hz. An input/output curve was generated by five different intensities. Then, the amplitude of PS and the slope of the fEPSP were measured. The lowest intensity of measurable responses was selected as Threshold intensity (T). Then other intensities consisting of 2×T to 5×T were tested. Stimulation amplitude that elicited 50–60% of the maximum response was used as a test pulse. After 20 minutes of stable baseline recording, the LTP-inducing protocol was applied and responses were recorded at 5, 15, 30, 45, and 60 minutes after LTP induction. Based on previous studies, LTP activity level changes at different time points (Bliss & Collingridge, 1993; Xin et al., 2006).

The current study investigated LTP induction level in different recording time points at 5, 15, 30, 45, and 60 minutes after the induction in order to determine the variations in the synaptic response of the CA1 of stratum pyramidal neurons. LTP was induced by High-Frequency Stimulation (HFS), which is a train of 100 stimuli at 100 Hz (Moradpour et al., 2016). The measured parameters were the slope of fEPSP and amplitudes of the PS recorded in the stratum pyramidal. The PS amplitude was measured as the difference between peak negativity and the middle of the first and second positivity.

Ampli5, ampli15, ampli30, ampli45, and ampli 60 represent PS amplitude at minutes 5, 15, 30, 45, and 60, respectively. The fEPSP slope was measured as the slope between the baseline and the peak of the first positive wave. Slop5, slop15, slop30, slop45, and slop60 denote the slopes of fEPSP at minutes 5, 15, 30, 45, and 60, respectively. The difference in fEPSP slope at minutes 60 and 5 (slopdiff60) and the difference in PS amplitude at minutes 60 and 5 (amplidiff60) were calculated in order to evaluate the velocity of LTP in certain periods of time.

LTP was quantified as the average percentage change in slope of fEPSP and the amplitude of PS. LTP taken during the last 10 minutes of each recording period after the LTP induction was compared with that of the fEPSP slope and the amplitude of PS taken during the last 10 minutes of baseline recording (Hosseinmardi et al., 2009).

#### 2.6.3. Serum total T4 and TSH measurement

All the samples were centrifuged (3000 g, 10 minutes, at 4 °C) and their serum was kept at −80 °C until the time of measurement. Serum total T4 was assayed using a commercial kit (Pishtaz Teb Zaman Co., Tehran, Iran). Their intra- and inter-assay coefficients of variation were 3.6%–5.8% and 4.4%–7.7%, respectively. Serum TSH was assayed using commercial ELISA (the enzyme-linked immunoassay) kit (EASTBIOPHARM, Hangzhou Eastbiopharm Co. Ltd., Zhejiang Province, China). The measurement ranges were from 0.1–30 mIU/mL and had a sensitivity of 0.05 mIU/mL.

#### 2.6.4. Statistical analysis

The statistical analysis was performed with SPSS V. 22.0. The distribution of data was checked by the Kolmogorov-Smirnoff test. The slope and amplitude at 5, 15, 30, 45, and 60 minutes were analyzed using four-way repeated measures ANOVA. In addition, slopdiff60, amplidiff60, slop60, and ampli 60 were analyzed using four-way ANONA followed by LSD post-hoc test for multiple comparisons. The analyses of TSH and total T4 between the two groups of the hypothyroid pups or dams as well as the corresponding control groups was evaluated by student t-test. The result was expressed as Mean±Standard Error of Mean (SEM) and differences were considered significant at P<0.05.

## 3. Results

### 3.1. Serum T4 and TSH levels in the control and TCH offspring rats

Serum levels of T4 and TSH in hypothyroid mothers and offspring are shown in Table 1 at the end of PTU treatment (PND 21). The analysis of data by t-test indicated that the serum levels of total T4 was significantly lower in hypothyroid mothers and offspring than the corresponding control groups on PND 21 (P=0.001). The analysis of serum levels of total T4 and TSH in TCH and control pups showed no significant differences at the end of the experiment (PND 48).

**Table 1.** Serum hormone levels of offspring and mothers at PND 21 and offspring at PND 48

**Groups**	**PND 21**				**PND 48**	

	**Mothers**		**Offspring**		**Offspring**	

	**T4, μg/dL**	**TSH, mIU/mL**	**T4**	**TSH**	**T4**	**TSH**
Control	6.75±0.24 ^*^	6.13±0.2 ^*^	6.61±0.19 ^*^	5.15±0.5 ^*^	6.19±0.14	5.23±0.16
TCH	2.01±0.32	30.12±2.5	1.76±0.3	26.03±4.2	5.6±0.28	5.52±0.18

Serum levels of hormones were measured at PND 21 both in offspring and mothers and in Offspring at PND 48; Data are expressed as Mean±SEM;

* P<0.001 compared with the Transient Congenital Hypothyroid (TCH) group; PND: Postnatal Day

### 3.2. Effects of treadmill exercise and sex hormones on PS amplitude and amplitude difference of LTP

**Figure 1.**
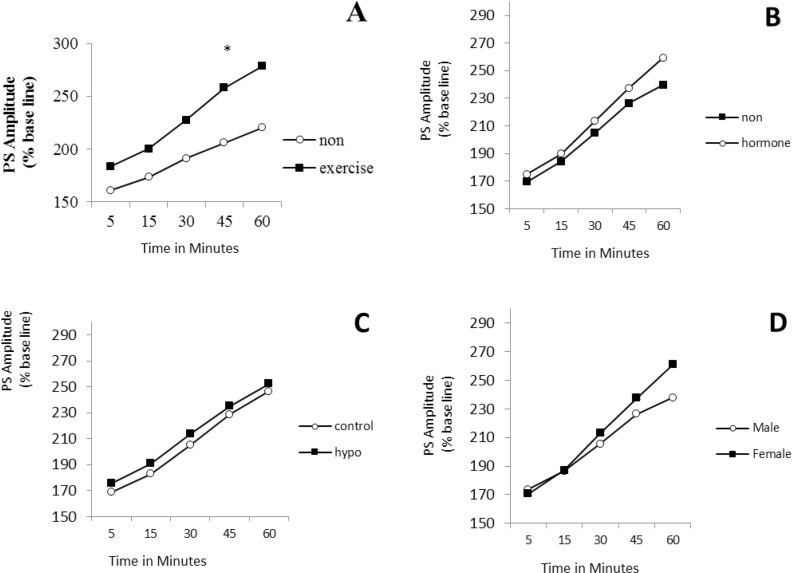
PS amplitude in CA1 of hippocampus in hypothyroid rats in 60 minutes after LTP induction A: Effects of treadmill exercise; B: Effects of hormone; C: Effects of hypothyroid; D: Effects of gender; The repeated measures ANOVA (4-way) revealed significant exect of exercise (P= 0.004); ^*^ indicates the significant effects of exercise compared with those of non-exersice.

**Figure 2.**
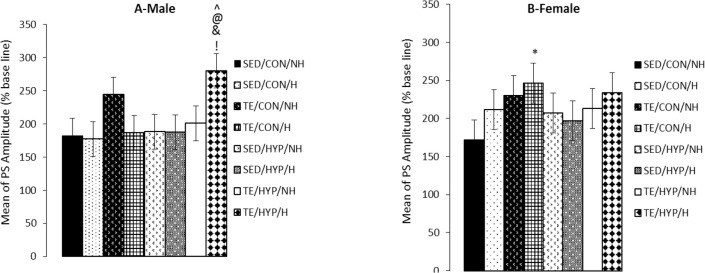
Effects of treadmill exercise and sex hormones on mean of PS amplitude (% baseline) in male (A) and female (B) rats of the control and TCH group during 60 minutes after HFs Panel A: mean of PS amplitude was significantly higher in TE/HYP/H subgroup compared to TE/CON/H, SED/HYP/NH, SED/HYP/H, and TE/HYP/NH subgroups. & indicates P=0.014, @ indicates P=0.015, ^ indicates P=0.014, and ! indicates P=0.036 compared with the TE/CON/H, SED/HYP/NH, SED/HYP/H and TE/HYP/NH subgroups, respectively; Panel B: mean of PS amplitude was significantly higher in TE/CON/H subgroup compared with SED/CON/NH subgroup. The results are expressed as Mean±SEM (4-way ANOVA); ^*^ P=0.047

A four-way repeated measures ANOVA (exercise×ho rmone×gender×hypothyroidism) on PS amplitude (at the time points of 5, 15, 30, 45, and 60 minutes after LTP induction) revealed the significant effect of exercise (F_1,80_=8.96, P=0.004), while the effects of gender, hormone, and hypothyroidism were not significant (Figure 1 A, B, C, and D). Post hoc comparisons showed that in male rats, the mean PS amplitude of the TE/HYP/H subgroup from time point 5 to time point 60 was significantly higher than those of TE/CON/H, SED/HYP/NH, SED/HYP/H, and TE/HYP/NH subgroups (P=0.014, P=0.015, P=0.014, and P=0.036, respectively) (Figure 2 A); and the mean PS amplitude of female rats in the TE/CON/H subgroup from time point 5 to time point 60 minutes was significantly higher than those of the SED/CON/NH subgroup (P=0.047) (Figure 2 B).

**Figure 3.**
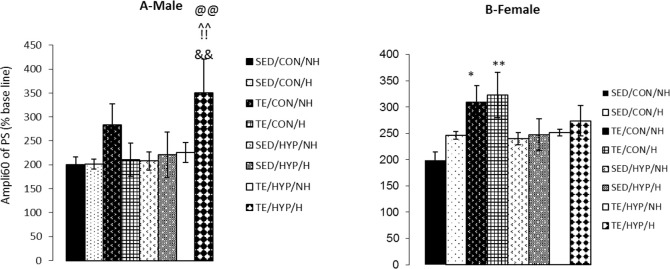
Effects of treadmill exercise and sex hormones on ampli 60 of PS (% baseline) in male (A) and female (B) rats of the control and TCH group at time point 60 minutes after HFs Panel A: Mean of ampli 60 of PS (% baseline) was significantly higher in TE/HYP/H subgroup compared to SED/HYP/H, SED/HYP/NH, TE/HYP/NH, and TE/CON/H subgroups. ^^^^ indicates P=0.006, @@ indicates P=0.002, !! indicates P=0.008, and && idicates P=0.003 compared to the SED/HYP/H, SED/HYP/NH, TE/HYP/NH and TE/CON/H subgroups, respectively; Panel B: Mean of ampli 60 of PS (% baseline) were significantly higher in TE/CON/H and TE/CON/NH subgroups compared with SED/CON/NH subgroup. The data are expressed as Mean±SEM (4-way ANOVA); ^*^ P=0.018; ^**^ P=0.008

A four-way ANOVA (exercise×hormone×gender×hy pothyroidism) on the PS amplitude of activity 60 minutes after LTP induction (ampli 60) demonstrated the significant effect of exercise (F_1,80_=12.96, P=0.001). Intergroup comparisons of the control female rats indicated that ampli 60 was significantly higher in TE/CON/H and TE/CON/NH subgroups than the SE/CON/NH subgroup (P=0.008 and P=0.018, respectively) (Figure 3 B). In the Intergroup comparisons of TCH male rats (but not female rats), ampli 60 was significantly higher in the TE/HYP/H subgroup than SED/HYP/H, SED/HYP/NH, and TE/HYP/NH subgroups (P=0.006, P=0.002, and P=0.008, respectively) (Figure 3 A). In the control group, comparison between the two genders indicated that the ampli 60 of the female rats in the TE/CON/H subgroup was significantly higher than that of the male rats in the corresponding subgroup (P=0.016) (Table 2). Moreover, intragroup comparisons of the control and TCH rats revealed that the ampli 60 of the male rats in the TE/HUP/H subgroup was significantly higher than that of the male rats in the TE/CON/H subgroup (P=0.003) (Figure 3 A).

**Table 2.** Mean ampli 60 in male and female pups

**Group**	**Ampli 60**	

	**Male**	**Female**
SED/CON/NH	200.52±15.57	198.38±16.12
SED/CON/H	201.47±10.81	246.04±7.10
TE/CON/NH	283.64±44.01	308.79±31.47
TE/CON/H	210.63±34.69	322.97±43.45 ^*^
SED/HYP/NH	208.24±18.94	239.57±11.29
SED/HYP/H	221.30±47.60	247.65±29.53
TE/HYP/NH	225.99±21.01	251.05±6.19
TE/HYP/H	350.67±70.67	273.85±29.21

* P<0.05 compared to male rats of the same subgroup on the ampli 60. Data are expressed as Mean±SEM.

**Figure 4.**
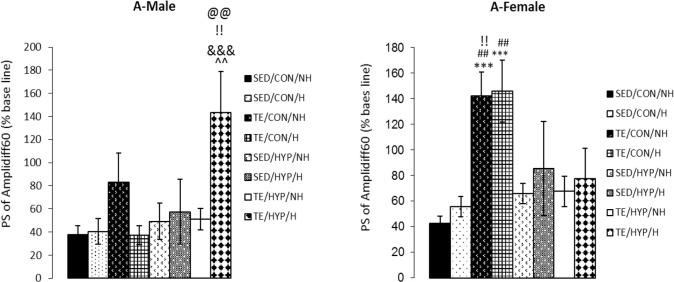
Effects of treadmill exercise and sex hormones on mean of PS amplidiff60 (PS amplitude difference of two time points of 5 and 60 minutes after HFs) (% baseline) in male (A) and female (B) rats of the control and TCH groups Panel A: mean of PS amplidiff60 (% baseline) was significantly higher in TE/HYP/H subgroup compared with SED/HYP/H, SED/HYP/NH, TE/HYP/NH, and TE/CON/H subgroups. ^^ indicates P=0.003, @@@ indicates P=0.001, !! indicates P=0.002, and &&& indicates P=0.001 compared with the SED/HYP/H, SED/HYP/NH, TE/HYP/NH, and TE/CON/H subgroups, respectively; Panel B: mean of PS amplidiff60 (% baseline) was significantly higher in TE/CON/NH and TE/CON/H subgroups compared to SED/CON/NH andSED/CON/H subgroups. ^***^ indicates P=0.001 and compared with the SED/CON/NH, ## indicates P=0.003 and P=0.002 compared with the SED/CON/H.As well, mean of PS of amplidiff60 (% baseline) was significantly higher in TE/CON/NH H subgroup compared with TE/HYP/NH; !! indicates P=0.010 compared with the TE/HYP/NH. The data are expressed as Mean±SEM (4-way ANOVA).

A four-way ANOVA (exercise×hormone×gender×hy pothyroidism) on PS amplitude difference between the two time points of 60 and 5 minutes after LTP induction (amplidiff60) demonstrated the significant effect of exercise (F_1,80_=15.34, P=0.001), gender (F_1,80_=5.27, P=0.024), gender×hypothyroidism (F_1,80_=5.73, P=0.019), and gender×hypothyroidism×exercise (F_1,80_=9.05, P=0.004). Intergroup comparisons of the control female rats indicated that the amplidiff60 of TE/CON/NH and TE/CON/H subgroups was significantly higher than that of the SED/CON/NH subgroup (P=0.001 in both cases), and the amplidiff60 of TE/CON/NH and TE/CON/H subgroups was significantly higher than that of the SED/CON/H subgroup (P=0.003 and P=0.002, respectively) (Figure 4 B).

Intergroup comparisons of TCH male rats (but not female rats) showed that amplidiff60 was significantly higher in the TE/HYP/H subgroup than SED/HYP/H, SED/HYP/NH, and TE/HYP/NH subgroups (P=0.003, P=0.001, and P=0.002, respectively) (Figure 4 A). In addition, comparison between the two genders indicated that in the control group, the amplidiff60 of TE/CON/NH and TE/CON/H subgroups of female rats was significantly higher than that of the corresponding male subgroups (P=0.040 and P=0.001, respectively) (Table 3).

**Table 3.** Mean amplidiff60 in male and female pups

**Group**	**Male**	**Female**

	**Amplidiff60**	**Amplidiff60**
SED/CON/NH	37.61±7.94	42.57±5.71
SED/CON/H	40.45±11.22	55.69±7.89
TE/CON/NH	83.14±24.97	142.16±18.64 ^*^
TE/CON/H	37.31±8.15	145.92±24.32 ^**^
SED/HYP/NH	49.26±15.75	65.87±7.73
SED/HYP/H	57.46±28	85.61±36.84
TE/HYP/NH	51.03±9.38	67.71±11.86
TE/HYP/H	143.20±35.73 ^*^	77.75±23.37

* P<0.05;

** Compared with male rats of the same subgroup on the amplidiff60. Data are expressed as Mean±SEM.

In TCH group, comparison between the two genders revealed that amplidiff60 was significantly higher in the TE/HYP/H subgroup of male rats than the corresponding females (P=0.023) (Table 3). Intra comparisons of the control and TCH rats demonstrated that amplidiff60 was significantly higher in the female rats of the TE/CON/NH subgroup than the female rats of the TE/HYP/NH subgroup (P=0.010) (Figure 4 B). Moreover, amplidiff60 was significantly higher in the male rats of the TE/HYP/H subgroup than the male rats of the TE/CON/H subgroup (P=0.001) (Figure 4 A).

### 3.3. Effects of treadmill exercise and sex hormones on the fEPSP slope of LTP

**Figure 5.**
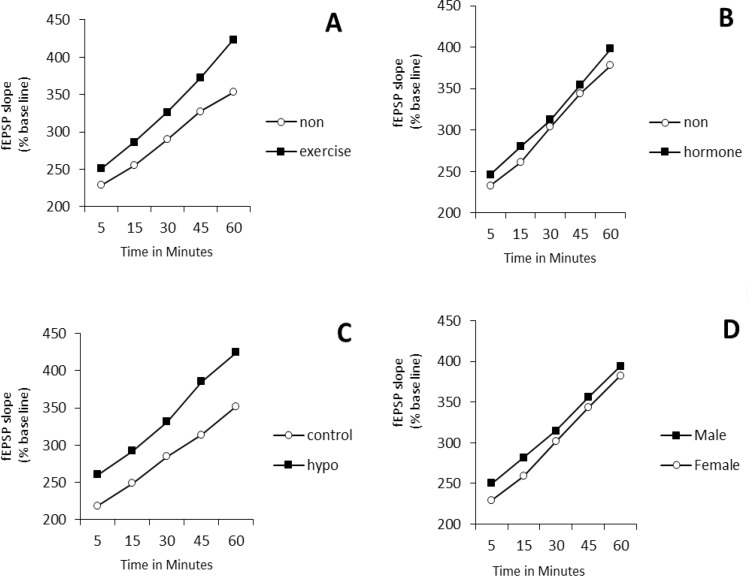
The fEPSP slope in CA1 of the hippocampus in hypothyroid rats in 60 minutes after LTP induction A) Effects of treadmill exercise; B) Effects of hormone; C) Effects of hypothyroid; D) Effects of gender; The repeated measurements ANOVA (4-way) revealed significant exect of hypothyroid (P= 0.039).

**Figure 6.**
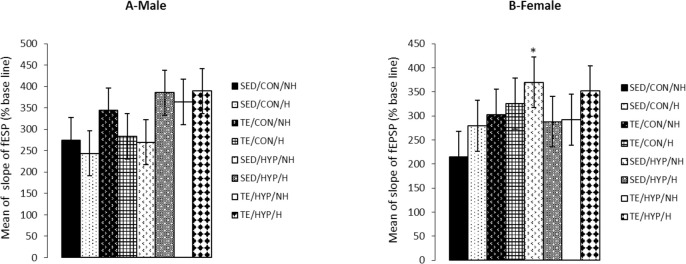
Effects of treadmill exercise and sex hormones on fEPSP slope (% baseline) in male (A) and female (B) rats of the control and TCH groups 60 minutes after HFs Panel A: Effects of treadmill exercise and sex hormones on mean of fEPSP slope in male rats were not significant; Panel B: mean of fEPSP slope was significantly higher in SED/HYP/NH subgroup compared with SED/CON/NH subgroup. The data are expressed as Mean±SEM (4-way ANOVA); ^*^ P=0.041

A four-way repeated measures ANOVA (exercise× hormone×gender×hypothyroidism) on the slope of fEPSP (at the time points of 5, 15, 30, 45, and 60 minutes) revealed the significant effect of hypothyroidism (F_1,80_=4.38, P=0.039), while the effects of gender and exercise were not significant (Figure 5). Post hoc comparisons showed that in female rats, the mean slope of fEPSP from time point 5 to time point 60 was significantly higher in the SED/HYP/NH subgroup than the SED/CON/NH subgroup (P=0.041) (Figure 6 B). However, there was no significant difference between the male rats (Figure 6 A).

**Figure 7.**
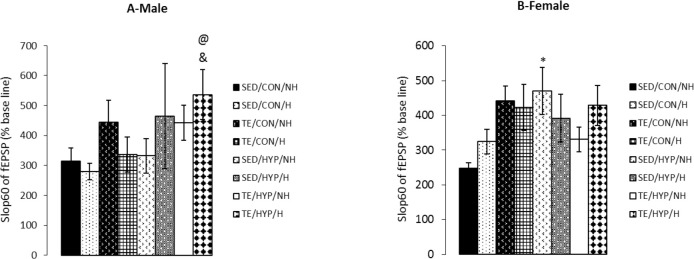
Effects of treadmill exercise and sex hormones on slop60 of fEPSP (% baseline) in male (A) and female (B) rats of control and TCH groups at 60 minutes after HFs Panel A: Mean of slop60 (% baseline) was significantly higher in TE/HYP/H subgroup compared to SED/HYP/NH and TE/CON/H sub-groups. @ indicates P=0.041 and & indicates P=0.046 compared with the SED/HYP/NH and TE/CON/H subgroups, respectively; Panel B: Mean of slop60 (% baseline) was significantly higher in SED/HYP/NH subgroup compared with SED/CON/NH subgroup. The data are expressed as Mean±SEM (4-way ANOVA); ^*^ P=0.027

A four-way ANOVA (exercise×hormone×gender×hyp othyroidism) on the slope of fEPSP 60 minutes after LTP induction (slop60) demonstrated the significant effect of exercise (F_1,80_=4.04, P=0.048) and hypothyroidism (F_1,80_=4.35, P=0.040). Intergroup comparisons of TCH male rats indicated that the slop60 of the TE/HYP/H subgroup was significantly higher than that of the SED/HYP/NH subgroup (P=0.041) (Figure 7 A). Intragroup comparisons of control and TCH rats showed that the slop60 of the female rats in the SED/HYP/NH subgroup was significantly higher than that of the female rats in the SED/CON/NH subgroup (P=0.027) (Figure 7 B). Furthermore, the slop60 of the male rats in the TE/HYP/H subgroup was significantly higher than that of the male rats in the TE/CON/H subgroup (P=0.046) (Figure 7 A).

**Figure 8.**
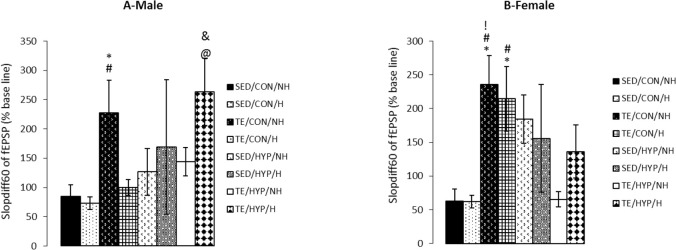
Effects of treadmill exercise and sex hormones on mean of slopdiff60 (sloe of fEPSP difference 5 and 60 minutes after HFs) (% baseline) in male (A) and female (B) rats of the control and TCH groups Panel A: Mean of slopdiff60 (% baseline) was significantly higher in TE/CON/NH subgroup compared with SED/CON/NH and SED/CON/H subgroups. ^*^ indicates P=0.038 and # indicates P=0.015, compared with the SED/CON/NH and SED/CON/H sub-groups, respectively; as well, the mean of slopdiff60 (% baseline) was significantly higher in TE/HYP/H subgroup compared to SED/HYP/NH and TE/CON/H sub-groups. @ indicates P=0.046 and & indicates P=0.018 compared with the SED/HYP/NH and TE/CON/H subgroups, respectively; Panel B: mean of slopdiff60 (% baseline) was significantly higher in TE/CON/H subgroup compared to SED/CON/NH and SED/CON/H subgroups. ^*^ indicates P=0.027 and # indicates P=0.026 compared to the SED/CON/NH and SED/CON/H subgroups respectively, as well, the mean of slopdiff60 (% baseline) was significantly higher in TE/CON/NH subgroup compared to SED/CON/NH, SED/CON/H and TE/HYP/NH sub-groups. ^*^ indicates P=0.012, # indicates P=0.012, and ! indicates P=0.018 compared to the SED/CON/NH, SED/CON/H, and TE/HYP/NH subgroups, respectively. The data are expressed as Mean±SEM (4-way ANOVA).

A four-way ANOVA (exercise×hormone×gender×hy pothyroidism) on the difference in the slope of fEPSP between 60 and 5 minutes after LTP induction (slopdiff60) demonstrated the significant effect of exercise (F_1,80_=6.01, P=0.016) as well as that of the interaction of exercise and hypothyroidism (F_1,80_=7.49, P=0.008). Intergroup comparisons of control female rats indicated that slopdiff60 was significantly higher in the TE/CON/NH subgroup than SED/CON/NH and SED/CON/H subgroups (P=0.012 in both cases), while slopdiff60 was significantly higher in the TE/CON/H subgroup than SED/CON/NH and SED/CON/H subgroups (P=0.027 and P=0.026, respectively) (Figure 8 B). Intergroup comparisons of control male rats indicated that the slopdiff60 of the TE/CON/NH subgroup was significantly higher than those of SED/CON/NH and SED/CON/H subgroups (P=0.038 and P=0.025, respectively) (Figure 8 A).

Intergroup comparisons of TCH male rats (but not female rats) showed that the slopdiff60 of the TE/HYP/H subgroup was significantly higher than that of the SED/HYP/NH subgroup (P=0.046) (Figure 8 A). Moreover, intragroup comparisons of control and TCH rats revealed that the slopdiff60 of the female rats in the TE/CON/NH subgroup was significantly higher than that of the female rats in the TE/HYP/NH subgroup (P=0.013) (Figure 8 B), while the slopdiff60 of the male rats in the TE/HYP/H subgroup was significantly higher than that of the male rats in the TE/CON/H subgroup (P=0.018) (Figure 8 A).

## 4. Discussion

Hypothyroidism during the critical periods of brain development impairs LTP induction (Dong et al., 2005). The relationship between learning/memory functions and LTP as the cellular basis has been proved (Bliss & Collingridge, 1993). Most of the negative effects of hypothyroidism are associated with changes in the hippocampus (Eichenbaum, 2004) .The current study aimed at investigating the possibility of an interaction between sex hormones and exercise to strengthen neural plasticity in TCH rats. The results demonstrated that although there was no significant difference in LTP between the male rats of control and TCH groups, an increase in fEPSP slope was observed among the female rats of the TCH group. The effects of exercise and/or hormone were different in both male and female groups. Exercise enhanced LTP in male and female rats of the control group, but the effect of hormone was not significant. The combination of exercise and testosterone (but none of them individually) enhanced LTP in TCH male rats, while the combination of exercise and estradiol or each of them individually did not produce such an effect on LTP in TCH female rats.

PTU as an anti-thyroid factor affects thyroid hormone secretion (Cooper et al., 1983). In the current study, PTU reduced the serum levels of T4 rats compared with the controls; and serum levels of TSH significantly increased in TCH rats compared with the controls. On PND 48, T4 and TSH levels were fully recovered in both male and female offspring, which meant animals were euthyroid at the time of test. Surprisingly, no difference was observed in fEPSP slope and PS amplitude between the sedentarynon-hormone subgroups of control and TCH male rats. Nevertheless, a significant increase in fEPSP slope and a non-significant increase in PS amplitude were observed in TCH female rats compared with the control female rats. Although mild TCH begins in late pregnancy (day 18 of gestation) and extends until the end of lactation, it does not affect fEPSP slope, but increases PS amplitude of LTP in adult rats (Gilbert, 2004).

The current study showed that hypothyroidism during the development differentially affects males and females with regards to hippocampal functions during adult-hood; thus, it has some long-term gender-specific functions. Multiple mechanisms may explain the variations observed in the slope of fEPSP and hippocampal function in TCH female rats. Some of these mechanisms include an overall increase in excitatory transmission that could involve excitatory glutamatergic receptors (An & Sun, 2017), an overall decrease in inhibitory transmission that could involve GABAergic receptors (Fasano et al., 2017), connectivity changes of excitatory projections from Schaeffer collaterals to CA1 (Onodera, Sato, & Kogure, 1986), or connectivity changes of regulatory inhibitory interneurons (Perez, Morin, & Lacaille, 2001).

These factors can make changes, individually or in combination, which contribute to the altered responses in adult females hippocampus treated prenatally and neonatally with PTU. Also, hippocampal formation yields a remarkable sensitivity to gonadal hormones, thereby explaining the existence of abundant reports on sexual differences in this region of the brain.

It can be approved that such differences are related to the presence of receptors for sexual steroids (Loy, Gerlach, & McEwen, 1988; Stumpf & Sar, 1977), since some hippocampal morphological and biochemical characteristics are liable to gonadal modulation (Harrelson & McEwen, 1987; Juraska et al., 1985). It remains to be exactly identified what makes males more sensitive to hypothyroid effects during this time period and whether or not there are other gender-specific outcomes following developmental hypothyroidism.

The current study found that exercise clearly affected the control group and enhanced LTP. These findings were in agreement with those of previous studies showing that treadmill exercise enhanced LTP in mice (Liu et al., 2011; Zhao et al., 2015). The results of the current study suggested that mild treadmill exercise was far less effective in male than female rats in the control group. It should be noted that exercise slightly, but significantly, increased LTP in male rats. It means that female rats were more sensitive to the potentiating effects of treadmill exercise than male ones, although voluntary exercise did not enhance LTP in female rats (Titterness et al., 2011). Moreover, in response to similar levels of exercises involving voluntary wheel running and forced treadmill running, female mice showed a greater capacity to increase their cardiac mass than males (Zhou et al., 2016). These contradictions may be due to variations in the type, length, and intensity of exercise trainings used.

In the current study, treadmill exercise did not enhance LTP in TCH rats. Frequent factors contribute to the useful effects of exercise on neuroplasticity in certain structures and resultant changes in learning and memory both in the healthy and the injured brains. Potential mechanisms may cause variations in the levels of growth factors, e g, Brain-Derived Neurotrophic Factor (BDNF) and Vascular Endothelial Growth Factor (VEGF), alterations to neurotransmitter and hormone signaling, as well as antiapoptotic activity and antioxidants (Cotman, Berchtold, & Christie, 2007). In the hippocampus, thyroid hormone deficiency decreases the mRNA expression of VEGF (Zhang, Blomgren, Kuhn, & Cooper-Kuhn, 2009) and BDNF (Abedelhaffez & Hassan, 2013). BDNF and VEGF are important factors in synaptic plasticity (Zhang et al., 2015) and their reduction may contribute to the inhibition of the effects of exercise on LTP and the adverse neurodevelopmental effects in hypothyroidism during pregnancy.

The other studies show that performing treadmill exercise during the postnatal period decreases the severity of hypothyroidism and results in the improvement of short-term memory and spatial learning ability (Shin et al., 2013) and the improvement of behavioral and biochemical disorders produced by maternal hypothyroidism in neonates (Shafiee et al., 2016). Anyway, changes in spatial learning such as the water maze test, which is a hippocampal-dependent task, are not necessarily associated with the ability to modify hippocampal synaptic strength (Del Olmo et al., 2006; Jeffery, 1997; Meiri et al., 1998).

In the current study, sex hormones did not enhance LTP either in the control or TCH group. Consistent with another study, testosterone administration in early puberty did not enhance LTP in male rats (Hebbard et al., 2003). Nevertheless, estrogen did not increase LTP induction, unexpectedly disagreeing with studies in which the induction of LTP increased by treatment with estradiol (Cordoba Montoya & Carrer, 1997; Inagaki et al., 2012). However, this result was consistent with that of a study in which estradiol chronic treatment had no significant effect on LTP (Barraclough, Ingram, & Brown, 1999). Exogenous estradiol effects can be observed while the environmental estradiol is removed before the treatment, i.e. after ovariectomy (Vierk, Brandt, & Rune, 2014). Although the current study did not measure serum levels of sex hormones, further studies are needed to use different doses of estradiol in the presence or absence of the environmental estradiol.

Combination effects of exercise and sex hormones also enhanced LTP in the control female and TCH male rats, but not in the control male or TCH female rats. No additional action was observed in the combined interventions in the control female rats. There was no significant difference between the two subgroups of TE/CON/H and TE/CON/NH in the control female rats, which was probably due to the effects of exercise itself; it might also be possible to assume that exercise treatment reached already a ceiling effect, which could not be further enhanced by estradiol. However, future studies are needed to apply more graded doses of estradiol and/or different intensities of exercises. However, in TCH male rats, the effect of exercise and sex hormone combination on LTP was significantly higher than the effect of the exercise or hormone alone. Also, exercise or hormone injection alone had no effects on LTP.

Moreover, aromatase activity is enhanced in TCH rats (Maran et al., 2000). It converts injected testosterone to estrogen and exerts its effects on the brain (Hojo et al., 2004; Kretz et al., 2004). In addition, according to the studies, androgens are synthesized in the hippocampus (Hojo et al., 2009). This local production can be applied by physiological stimuli such as exercise (Okamoto et al., 2012). Mild treadmill exercise results in increased hippocampal dihydrotestosterone levels, and androgens stimulate exercise-induced hippocampal neurogenesis (65). On the other hand, exercise gives the brain benefit of VEGF and BDNF (van Praag, 2008).

VEGF directly increases neurogenesis (Jin et al., 2002; Kirby, Kuwahara, Messer, & Wyss-Coray, 2015). Treatment with testosterone also stimulates the production of VEGF in canary and protein levels, while BDNF mRNA expression in the Higher Vocal Center (HVC) is increased after treatment with testosterone (Louissaint et al., 2002). The effects of BDNF are examined on synaptic promotion (Leal et al., 2015). VEGF is one of the important factors for LTP induction in the hippocampus (Zhang et al., 2015). Therefore, exercise and testosterone via VEGF and BDNF can have a common and synergistic pathway for LTP induction. Not surprisingly, these combined effects were not observed with LTP induction in the control group due to the hypothesis that an optimal level of testosterone is necessary for brain organization well suited for LTP induction (Holland, Bandelow, & Hogervorst, 2011).

To summerize, the decrease in thyroid hormones, beginning in early pregnancy and continuing until the end of it, can cause changes in synaptic plasticity in slices taken from young animals. Despite the return of thyroid hormone to normal levels in TCH rats, their response to high-frequency stimuli in LTP was different from that of control rats; nevertheless, the effects of exercise were not observed in them and exercise had no effects on LTP induction, while male TCH rats responded to the combinatorial model of exercise and testosterone.

## Ethical Considerations

### Compliance with ethical guidelines

All experimental protocols were undertaken in accordance with the guidelines of the 1975 Declaration of Helsinki, as reflected in the guidelines of the Medical Ethics Committee, Ministry of Health, Iran. In addition, the Regional Medical Ethics Committee in West Azerbaijan Province, Iran, approved the study (Ethical code No.: 93.470253).

## References

[B1] Abedelhaffez A. S. Hassan A. (2013). Brain derived neurotrophic factor and oxidative stress index in pups with developmental hypothyroidism: Neuroprotective effects of selenium. Acta Physiologica Hungarica, 100(2), 197–210. [DOI:10.1556/APhysiol.100.2013.2.7] [PMID ] 23708947

[B2] An L. Sun W. (2017). Prenatal melamine exposure impairs spatial cognition and hippocampal synaptic plasticity by presynaptic and postsynaptic inhibition of glutamatergic transmission in adolescent offspring. Toxicology Letters, 269, 55–64. [DOI:10.1016/j.toxlet.2017.02.005] [PMID ] 28185983

[B3] Barraclough D. J. Ingram C. D. Brown M. W. (1999). Chronic treatment with oestradiol does not alter in vitro LTP in subfield CA1 of the female rat hippocampus. Neuropharmacology, 38(1), 65–71. [DOI:10.1016/S0028-3908(98)00157-9] 10193899

[B4] Berchtold N. C. Castello N. Cotman C. W. (2010). Exercise and time-dependent benefits to learning and memory. Neuroscience, 167(3), 588–97. [DOI:10.1016/j.neuroscience.2010.02.050] [PMID ] [PMCID ] 20219647 PMC2857396

[B5] Bernal J. (2002). Action of thyroid hormone in brain. Journal of Endocrinological Investigation, 25(3), 268–88. [DOI:10.1007/BF03344003] [PMID ] 11936472

[B6] Bhavani N. (2011). Transient congenital hypothyroidism. Indian Journal of Endocrinology and Metabolism, 15(Suppl.2), S117–20. [DOI:10.4103/2230-8210.83345] [PMID ] [PMCID ] 21966647 PMC3169860

[B7] Bleichrodt N. Shrestha R. M. West C. E. Hautvast J. G. van de Vijver F. J. Born M. P. (1996). The benefits of adequate iodine intake. Nutrition Reviews, 54(4), S72–78. [DOI:10.1111/j.1753-4887.1996.tb03901.x] 8700456

[B8] Bliss T. V. Collingridge G. L. (1993). A synaptic model of memory: Long-Term Potentiation in the hippocampus. Nature, 361(6407), 31–9. [DOI:10.1038/361031a0] [PMID ] 8421494

[B9] Calaciura F. Mendorla G. Distefano M. Castorina S. Fazio T. Motta R. M. (1995). Childhood IQ measurements in infants with transient congenital hypothyroidism. Clinical Endocrinology, 43(4), 473–7. [DOI:10.1111/j.1365-2265.1995.tb02620.x] 7586623

[B10] Chiao Y. C. Lee H. Y. Wang S. W. Hwang J. J. Chien C. H. Huang S. W. (1999). Regulation of thyroid hormones on the production of testosterone in rats. Journal of Cellular Biochemistry, 73(4), 554–62. [DOI:10.1002/(SICI)1097-4644(19990615)73:43.0.CO;2-L] 10733348

[B11] Ciloglu F. Peker I. Pehlivan A. Karacabey K. Ilhan N. Saygin O. (2005). Exercise intensity and its effects on thyroid hormones. Neuroendocrinology Letters, 26(6), 830–4. [PMID ] 16380698

[B12] Collaer M. L. Hines M. (1995). Human behavioral sex differences: A role for gonadal hormones during early development? Psychological Bulletin, 118(1), 55–107. [DOI:10.1037/0033-2909.118.1.55] [PMID ] 7644606

[B13] Cooper D. S. Kieffer J. D. Halpern R. Saxe V. Mover H. Maloof F. (1983). Propylthiouracil (PTU) pharmacology in the rat. II; Effects of PTU on thyroid function. Endocrinology, 113(3), 921–8. [DOI:10.1210/endo-113-3-921] [PMID ] 6872961

[B14] Cordoba Montoya D. A. Carrer H. F. (1997). Estrogen facilitates induction of long term potentiation in the hippocampus of awake rats. Brain Research, 778(2), 430–8. [PMID ] 9459564 10.1016/s0006-8993(97)01206-7

[B15] Cotman C. W. Berchtold N. C. Christie L. A. (2007). Exercise builds brain health: Key roles of growth factor cascades and inflammation. Trends in Neurosciences, 30(9), 464–72. [DOI:10.1016/j.tins.2007.06.011] [PMID ] 17765329

[B16] Del Olmo N. Higuera-Matas A. Miguens M. Garcia-Lecumberri C. Borcel E. Solis J. M. (2006). Hippocampal synaptic plasticity and water maze learning in cocaine self-administered rats. Annals of the New York Academy of Sciences, 1074(1), 427–37. [DOI:10.1196/annals.1369.043] [PMID ] 17105941

[B17] Dong J. Yin H. Liu W. Wang P. Jiang Y. Chen J. (2005). Congenital iodine deficiency and hypothyroidism impair LTP and decrease C-fos and C-jun expression in rat hippocampus. NeuroToxicology, 26(3), 417–26. [DOI:10.1016/j.neuro.2005.03.003] [PMID ] 15935212

[B18] Ebrahimi L. Saboory E. Roshan-Milani S. Hashemi P. (2014). Effect of prenatal forced-swim stress and morphine co-administration on pentylentetrazol-induced epileptic behaviors in infant and prepubertal rats. Developmental Psychobiology, 56(6), 1179–86. [DOI:10.1002/dev.21198] 24464467

[B19] Eddy M. C. Rifken K. M. Toufexis D. J. Green J. T. (2013). Gonadal hormones and voluntary exercise interact to improve discrimination ability in a set-shift task. Behavioral Neuroscience, 127(5), 744–54. [DOI:10.1037/a0033728] [PMID ] [PMCID ] 23978149 PMC3970407

[B20] Eichenbaum H. (2004). Hippocampus: cognitive processes and neural representations that underlie declarative memory. Neuron, 44(1), 109–20. [DOI:10.1016/j.neuron.2004.08.028] [PMID ] 15450164

[B21] Fasano C. Rocchetti J. Pietrajtis K. Zander J. F. Manseau F. Sakae D. Y. (2017). Regulation of the hippocampal network by VGLUT3-positive CCK-GABAergic basket cells. Frontiers in Cellular Neuroscience, 11(140), 1–15. [DOI:10.3389/fncel.2017.00140] 28559797 PMC5432579

[B22] Fester L. Prange-Kiel J. Jarry H. Rune G. M. (2011). Estrogen synthesis in the hippocampus. Cell and Tissue Research, 345(3), 285–94. [DOI:10.1007/s00441-011-1221-7] [PMID ] 21826442

[B23] Gerges N. Z. Alkadhi K. A. (2004). Hypothyroidism impairs late LTP in CA1 region but not in dentate gyrus of the intact rat hippocampus: MAPK involvement. Hippocampus, 14(1), 40–5. [DOI:10.1002/hipo.10165] [PMID ] 15058481

[B24] Gilbert M. E. (2004). Alterations in synaptic transmission and plasticity in area CA1 of adult hippocampus following developmental hypothyroidism. Developmental Brain Research, 148(1), 11–8. [DOI:10.1016/j.devbrainres.2003.09.018] [PMID] 14757514

[B25] Gottesfeld Z. Butler I. J. Findley W. E. (1985). Prenatal and postnatal hypothyroidism abolishes lesion-induced noradrenergic sprouting in the adult rat. Journal of Neuroscience Research, 14(1), 61–9. [DOI:10.1002/jnr.490140106] [PMID] 4020898

[B26] Haimov-Kochman R. Berger I. (2014). Cognitive func tions of regularly cycling women may differ throughout the month, depending on sex hormone status; a possible explanation to conflicting results of studies of ADHD in females. Frontiers in Human Neuroscience, 8(191), 1–6. [DOI:10.3389/fnhum.2014.00191] [PMID] [PMCID] 24744721 PMC3978296

[B27] Harrelson A. McEwen B. (1987). Gonadal steroid modulation of neurotransmitter-stimulated cAMP accumulation in the hippocampus of the rat. Brain Research, 404(1–2), 89–94. [DOI:10.1016/0006-8993(87)91358-8] 2882811

[B28] Hashemi P. Ebrahimi L. Saboory E. Roshan-Milani S. (2013). Effect of restraint stress during gestation on pentylenetetrazol-induced epileptic behaviors in rat offspring. Iranian Journal of Basic Medical Sciences, 16(9), 979–84. [PMID] [PMCID] 24171076 PMC3804846

[B29] Hebbard P. C. King R. R. Malsbury C. W. Harley C. W. (2003). Two organizational effects of pubertal testosterone in male rats: transient social memory and a shift away from Long-Term Potentiation following a tetanus in hippocampal CA1. Experimental Neurology, 182(2), 470–5. [DOI:10.1016/S0014-4886(03)00119-5] 12895458

[B30] Hojo Y. Hattori T. A. Enami T. Furukawa A. Suzuki K. Ishii H. T. (2004). Adult male rat hippocampus synthesizes estradiol from pregnenolone by cytochromes P45017alpha and P450 aromatase localized in neurons. Proceedings of the National Academy of Sciences of the United States of America, 101(3), 865–70. [DOI:10.1073/pnas.2630225100] [PMID] [PMCID] 14694190 PMC321772

[B31] Hojo Y. Higo S. Ishii H. Ooishi Y. Mukai H. Murakami G. (2009). Comparison between hippocampus-synthesized and circulation-derived sex steroids in the hippocampus. Endocrinology, 150(11), 5106–12. [DOI:10.1210/en.2009-0305] [PMID] 19589866

[B32] Holland J. Bandelow S. Hogervorst E. (2011). Testosterone levels and cognition in elderly men: A review. Maturitas, 69(4), 322–37. [DOI:10.1016/j.maturitas.2011.05.012] [PMID] 21696899

[B33] Hosseinmardi N. Fathollahi Y. Naghdi N. Javan M. (2009). Theta pulse stimulation: A natural stimulus pattern can trigger long-term depression but fails to reverse Long-Term Potentiation in morphine withdrawn hippocampus area CA1. Brain Research, 1296, 1–14. [DOI:10.1016/j.brainres.2009.08.020] [PMID] 19686713

[B34] Inagaki T. Kaneko N. Zukin R. S. Castillo P. E. Etgen A. M. (2012). Estradiol attenuates ischemia-induced death of hippocampal neurons and enhances synaptic transmission in aged, long-term hormone-deprived female rats. PLoS One, 7(6), e38018 [DOI:10.1371/journal.pone.0038018] [PMID] [PMCID] 22675505 PMC3366987

[B35] Jeffery K. J. (1997). LTP and spatial learning-where to next. Hippocampus, 7(1), 95–110. [DOI:10.1002/(SICI)1098-1063(1997)7:13.0.CO;2-D] 9138673

[B36] Jin K. Zhu Y. Sun Y. Mao X. O. Xie L. Greenberg D. A. (2002). Vascular Endothelial Growth Factor (VEGF) stimulates neurogenesis in vitro and in vivo. Proceedings of the National Academy of Sciences of the United States of America, 99(18), 11946–50. [DOI:10.1073/pnas.182296499] [PMID] [PMCID] 12181492 PMC129374

[B37] Juraska J. M. Fitch J. M. Henderson C. Rivers N. (1985). Sex differences in the dendritic branching of dentate granule cells following differential experience. Brain Research, 333(1), 73–80. [DOI:10.1016/0006-8993(85)90125-8] 3995290

[B38] Kim D. H. Ko I. G. Kim B. K. Kim T. W. Kim S. E. Shin M. S. (2010). Treadmill exercise inhibits traumatic brain injury-induced hippocampal apoptosis. Physiology & Behavior, 101(5), 660–5. [DOI:10.1016/j.physbeh.2010.09.021] [PMID] 20888848

[B39] Kirby E. D. Kuwahara A. A. Messer R. L. Wyss-Coray T. (2015). Adult hippocampal neural stem and progenitor cells regulate the neurogenic niche by secreting VEGF. Proceedings of the National Academy of Sciences of the United States of America, 112(13), 4128–33. [DOI:10.1073/pnas.1422448112] [PMID] [PMCID] 25775598 PMC4386397

[B40] Klubo-Gwiezdzinska J. Burman K. D. Van Nostrand D. Wartofsky L. (2011). Levothyroxine treatment in pregnancy: indications, efficacy, and therapeutic regimen. Journal of Thyroid Research, 2011(843591), 1–12. [DOI:10.4061/2011/843591] PMC3163038 21876837

[B41] Korenchevsky V. Hall K. (1941). Correlation between sex hormones, thyroid hormones and desoxycorticosterone as judged by their effects on the weights of organs of gonadectomized rats. Biochemical Journal, 35(5–6), 726–35. [DOI:10.1042/bj0350726] PMC1265550 16747441

[B42] Krassas G. E. (2000). Thyroid disease and female reproduction. Fertility and Sterility, 74(6), 1063–70. [DOI:10.1016/S0015-0282(00)01589-2] 11119728

[B43] Kretz O. Fester L. Wehrenberg U. Zhou L. Brauckmann S. Zhao S. (2004). Hippocampal synapses depend on hippocampal estrogen synthesis. Journal of Neuroscience, 24(26), 5913–21. [DOI:10.1523/JNEUROSCI.5186-03.2004] [PMID] 15229239 PMC6729232

[B44] Lasley S. M. Gilbert M. E. (2011). Developmental thyroid hormone insufficiency reduces expression of Brain-Derived Neurotrophic Factor (BDNF) in adults but not in neonates. Neurotoxicology and Teratology, 33(4), 464–72. [DOI:10.1016/j.ntt.2011.04.001] [PMID ] 21530650

[B45] Leal G. Afonso P. M. Salazar I. L. Duarte C. B. (2015). Regulation of hippocampal synaptic plasticity by BDNF. Brain Research, 1621, 82–101. [DOI:10.1016/j.brainres.2014.10.019] [PMID ] 25451089

[B46] Li C. H. Wang S. Z. Cai Z. L. Liu W. X. Xu S. T. Xiao P. (2012). Effects of intrahippocampal L-NAME treatment on the behavioral Long-Term Potentiation in dentate gyrus. Neuroscience Letters, 528(2), 201–4. [DOI:10.1016/j.neulet.2012.08.056] [PMID ] 22982147

[B47] Liu H. L. Zhao G. Cai K. Zhao H. H. Shi L. D. (2011). Treadmill exercise prevents decline in spatial learning and memory in APP/PS1 transgenic mice through improvement of hippocampal Long-Term Potentiation. Behavioural Brain Research, 218(2), 308–14. [DOI:10.1016/j.bbr.2010.12.030] [PMID ] 21192984

[B48] Liu Y. F. Chen H. I. Wu C. L. Kuo Y. M. Yu L. Huang A. M. (2009). Differential effects of treadmill running and wheel running on spatial or aversive learning and memory: roles of amygdalar brain-derived neurotrophic factor and synaptotagmin I. The Journal of Physiology, 587(Pt.13), 3221–31. [DOI:10.1113/jphysiol.2009.173088] [PMID ] [PMCID ] 19451201 PMC2727033

[B49] Louissaint A. Jr. Rao S. Leventhal C. Goldman S. A. (2002). Coordinated interaction of neurogenesis and angiogenesis in the adult songbird brain. Neuron, 34(6), 945–60. [DOI:10.1016/S0896-6273(02)00722-5] 12086642

[B50] Loy R. Gerlach J. L. McEwen B. S. (1988). Autoradiographic localization of estradiol-binding neurons in the rat hippocampal formation and entorhinal cortex. Brain Research, 467(2), 245–51. [DOI:10.1016/0165-3806(88)90028-4] 3378173

[B51] Maran R. R. Arunakaran J. Jeyaraj D. A. Ravichandran K. Ravisankar B. Aruldhas M. M. (2000). Transient neonatal hypothyroidism alters plasma and testicular sex steroid concentration in puberal rats. Endocrine Research, 26(3), 411–29. [DOI:10.3109/07435800009066177] [PMID ] 11019905

[B52] McEwen B. S. (2001). Invited review: Estrogens effects on the brain: Multiple sites and molecular mechanisms. Journal of Applied Physiology, 91(6), 2785–801. [DOI:10.1152/jappl.2001.91.6.2785] [PMID ] 11717247

[B53] Meiri N. Sun M. K. Segal Z. Alkon D. L. (1998). Memory and Long-Term Potentiation (LTP) dissociated: Normal spatial memory despite CA1 LTP elimination with Kv1.4 antisense. Proceedings of the National Academy of Sciences of the United States of America, 95(25), 15037–42. [DOI:10.1073/pnas.95.25.15037] [PMID ] 9844011 PMC24571

[B54] Moradpour F. Fathollahi Y. Naghdi N. Hosseinmardi N. Javan M. (2016). Prepubertal castration-associated developmental changes in sigma-1 receptor gene expression levels regulate hippocampus area CA1 activity during adolescence. Hippocampus, 26(7), 933–46. [DOI:10.1002/hipo.22576] [PMID ] 26860755

[B55] Nabavi S. Fox R. Proulx C. D. Lin J. Y. Tsien R. Y. Malinow R. (2014). Engineering a memory with LTD and LTP. Nature, 511(7509), 348–52. [DOI:10.1038/nature13294] [PMID ] [PMCID ] 24896183 PMC4210354

[B56] Okamoto M. Hojo Y. Inoue K. Matsui T. Kawato S. McEwen B. S. (2012). Mild exercise increases dihydrotestosterone in hippocampus providing evidence for androgenic mediation of neurogenesis. Proceedings of the National Academy of Sciences of the United States of America, 109(32), 13100–5. [DOI:10.1073/pnas.1210023109] [PMID ] [PMCID ] 22807478 PMC3420174

[B57] Onodera H. Sato G. Kogure K. (1986). Lesions to Schaffer collaterals prevent ischemic death of CA1 pyramidal cells. Neuroscience Letters, 68(2), 169–74. [DOI:10.1016/0304-3940(86)90136-9] 2875420

[B58] Panahi Y. Saboory E. Rassouli A. Sadeghi-Hashjin G. Roshan-Milani S. Derafshpour L. (2017). The effect of selective opioid receptor agonists and antagonists on epilepti-form activity in morphine-dependent infant mice hippocampal slices. International Journal of Developmental Neuroscience, 60, 56–62. [DOI:10.1016/j.ijdevneu.2017.04.003] [PMID ] 28455226

[B59] Perez Y. Morin F. Lacaille J. C. (2001). A hebbian form of Long-Term Potentiation dependent on mGluR1a in hippocampal inhibitory interneurons. Proceedings of the National Academy of Sciences, 98(16), 9401–6. [DOI:10.1073/pnas.161493498] [PMID ] [PMCID ] PMC55433 11447296

[B60] Shafiee S. M. Vafaei A. A. Rashidy-Pour A. (2016). Effects of maternal hypothyroidism during pregnancy on learning, memory and hippocampal BDNF in rat pups: Beneficial effects of exercise. Neuroscience, 329, 151–61. [DOI:10.1016/j.neuroscience.2016.04.048] [PMID ] 27181637

[B61] Shin M. S. Ko I. G. Kim S. E. Kim B. K. Kim T. S. Lee S. H. (2013). Treadmill exercise ameliorates symptoms of methimazole-induced hypothyroidism through enhancing neurogenesis and suppressing apoptosis in the hippocampus of rat pups. International Journal of Developmental Neuroscience, 31(3), 214–23. [DOI:10.1016/j.ijdevneu.2013.01.003] [PMID ] 23328696

[B62] Smith M. D. Jones L. S. Wilson M. A. (2002). Sex differences in hippocampal slice excitability: Role of testosterone. Neuroscience, 109(3), 517–30. [DOI:10.1016/S0306-4522(01)00490-0] 11823063

[B63] Stumpf W. E. Sar M. (1977). Steroid hormone target cells in the periventricular brain: Relationship to peptide hormone producing cells. Federation Proceedings, 36(7), 1973–7. [PMID ] 324816

[B64] Talsness C. E. Kuriyama S. N. Sterner-Kock A. Schnitker P. Grande S. W. Shakibaei M. (2008). In-utero and lactational exposures to low doses of polybrominated diphenyl ether-47 alter the reproductive system and thyroid gland of female rat offspring. Environmental Health Perspectives, 116(3), 308–14. [DOI:10.1289/ehp.10536] [PMID ] [PMCID ] 18335096 PMC2265047

[B65] Tavassoli E. Saboory E. Teshfam M. Rasmi Y. Roshan-Milani S. Ilkhanizadeh B. (2013). Effect of prenatal stress on density of NMDA receptors in rat brain. International Journal of Developmental Neuroscience, 31(8), 790–5. [DOI:10.1016/j.ijdevneu.2013.09.010] [PMID ] 24120877

[B66] Titterness A. K. Wiebe E. Kwasnica A. Keyes G. Christie B. R. (2011). Voluntary exercise does not enhance Long-Term Potentiation in the adolescent female dentate gyrus. Neuroscience, 183, 25–31. [DOI:10.1016/j.neuroscience.2011.03.050] [PMID ] 21458541

[B67] van Praag H. (2008). Neurogenesis and exercise: Past and future directions. Neuromolecular Medicine, 10(2), 128–40. [DOI:10.1007/s12017-008-8028-z] [PMID ] 18286389

[B68] van Praag H. Shubert T. Zhao C. Gage F. H. (2005). Exercise enhances learning and hippocampal neurogenesis in aged mice. Journal of Neuroscience, 25(38), 8680–5. [DOI:10.1523/JNEUROSCI.1731-05.2005] [PMID ] [PMCID ] 16177036 PMC1360197

[B69] Vierk R. Brandt N. Rune G. M. (2014). Hippocampal estradiol synthesis and its significance for hippocampal synaptic stability in male and female animals. Neuroscience, 274, 24–32. [DOI:10.1016/j.neuroscience.2014.05.003] [PMID ] 24846612

[B70] Whitlock J. R. Heynen A. J. Shuler M. G. Bear M. F. (2006). Learning induces Long-Term Potentiation in the hippocampus. Science, 313(5790), 1093–7. [DOI:10.1126/science.1128134] [PMID ] 16931756

[B71] Williams G. R. (2008). Neurodevelopmental and neurophysiological actions of thyroid hormone. Journal of Neuroendocrinology, 20(6), 784–94. [DOI:10.1111/j.1365-2826.2008.01733.x] [PMID ] 18601701

[B72] Xin W. J. Gong Q. J. Xu J. T. Yang H. W. Zang Y. Zhang T. (2006). Role of phosphorylation of ERK in induction and maintenance of LTP of the C-fiber evoked field potentials in spinal dorsal horn. Journal of Neuroscience Research, 84(5), 934–43. [DOI:10.1002/jnr.21013] [PMID ] 16902997

[B73] Zhang L. Blomgren K. Kuhn H. G. Cooper-Kuhn C. M. (2009). Effects of postnatal thyroid hormone deficiency on neurogenesis in the juvenile and adult rat. Neurobiology of Disease, 34(2), 366–74. [DOI:10.1016/j.nbd.2009.02.006] [PMID ] 19233274

[B74] Zhang N. Xing M. Wang Y. Tao H. Cheng Y. (2015). Repetitive transcranial magnetic stimulation enhances spatial learning and synaptic plasticity via the VEGF and BDNF-NM-DAR pathways in a rat model of vascular dementia. Neuroscience, 311, 284–91. [DOI:10.1016/j.neuroscience.2015.10.038] [PMID ] 26518460

[B75] Zhao G. Liu H. L. Zhang H. Tong X. J. (2015). Treadmill exercise enhances synaptic plasticity, but does not alter beta-amyloid deposition in hippocampi of aged APP/PS1 transgenic mice. Neuroscience, 298, 357–66. [DOI:10.1016/j.neuroscience.2015.04.038] [PMID ] 25917310

[B76] Zhou Y. Zhao M. Zhou C. Li R. (2016). Sex differences in drug addiction and response to exercise intervention: From human to animal studies. Frontiers in neuroendocrinology, 40, 24–41. [DOI:10.1016/j.yfrne.2015.07.001] [PMID ] [PMCID ] 26182835 PMC4712120

